# Diagnosis of Carcinogenic Pathologies through Breath Biomarkers: Present and Future Trends

**DOI:** 10.3390/biomedicines11113029

**Published:** 2023-11-11

**Authors:** Valentina Vassilenko, Pedro Catalão Moura, Maria Raposo

**Affiliations:** Laboratory for Instrumentation, Biomedical Engineering and Radiation Physics (LIBPhys-UNL), Department of Physics, NOVA School of Science and Technology, NOVA University of Lisbon, Campus FCT-UNL, 2829-516 Caparica, Portugal; mfr@fct.unl.pt

**Keywords:** carcinogenic diseases, cancer, biomarkers, volatile organic compounds (VOCs), exhaled air, breath analysis

## Abstract

The assessment of volatile breath biomarkers has been targeted with a lot of interest by the scientific and medical communities during the past decades due to their suitability for an accurate, painless, non-invasive, and rapid diagnosis of health states and pathological conditions. This paper reviews the most relevant bibliographic sources aiming to gather the most pertinent volatile organic compounds (VOCs) already identified as putative cancer biomarkers. Here, a total of 265 VOCs and the respective bibliographic sources are addressed regarding their scientifically proven suitability to diagnose a total of six carcinogenic diseases, namely lung, breast, gastric, colorectal, prostate, and squamous cell (oesophageal and laryngeal) cancers. In addition, future trends in the identification of five other forms of cancer, such as bladder, liver, ovarian, pancreatic, and thyroid cancer, through perspective volatile breath biomarkers are equally presented and discussed. All the results already achieved in the detection, identification, and quantification of endogenous metabolites produced by all kinds of normal and abnormal processes in the human body denote a promising and auspicious future for this alternative diagnostic tool, whose future passes by the development and employment of newer and more accurate collection and analysis techniques, and the certification for utilisation in real clinical scenarios.

## 1. Introduction

During recent decades, carcinogenic diseases have been among the most common and lethal pathologies. The American Cancer Society estimates that around 1.9 million new cases of cancer and 0.6 million deaths happen every year, just in the United States of America [1,2]. For comparison, the same statistical data from ten years ago, i.e., 2013, shows around 1.6 million new cases and 0.58 million deaths registered in the USA [3]. This represents an increase of 18% and 3%, respectively. These numbers are equivalent for the rest of the world, especially for the so-called developed countries. So, the impact of carcinogenic pathologies on society and public health is evident and concerning.

The methodologies for diagnosing and treating new cases of cancer have been developing and evolving, which should be translatable in a decrease in both the number of cases and deaths. Nonetheless, these values have been stabilizing and even tending to rise, at least during the past ten years [4]. The success of the treatment of a carcinogenic pathology depends on several factors; the stage in which the disease is detected is one of those very important factors for achieving a successful cure since pathologies detected in later stages are often more lethal. In fact, the sooner the health condition is detected and accurately diagnosed, the higher the chance of a cure and survival. Most conventional diagnostic procedures, however, are often time-consuming and tend to delay the beginning of the clinical treatment. These interventions, which range from simple blood analyses to complex biopsies, are significantly invasive and often imply high levels of discomfort and pain. Therefore, a fast, accurate and painless diagnosis and treatment methodologies are in high demand for medical purposes.

The assessment and quantification of the metabolites produced by all kinds of normal and abnormal processes in the human body can be an informative source of information about the organism and its health condition [5]. In addition, the non-invasive, painless, and low-cost character of the exhaled breath analysis has placed this diagnosis procedure among the most promising ones and with the potential to tackle all the current challenges of the contemporary diagnostic procedures addressed above [6].

The great advances that mark the history of breath analysis arise from the revolutionary work of Pauling et al. (1971) [7], who detected the existence of a high number of volatile organic compounds (>200) present in exhaled air through gas chromatography. Breath analysis has expanded since then, mainly in the detection and validation of potential sets of biomarkers associated with several diseases, including but not restricted to the oncological ones [8,9]. A biomarker is defined by the National Cancer Institute as a “biological molecule found in blood, body fluids, or tissues that is a sign of a normal or abnormal process, health condition, or disease” [10]. Their usefulness for the assessment of cancer pathologies, however, has not been restricted to diagnosis. In fact, biomarkers have been addressed and deeply studied regarding their suitability for risk assessment, health state screening, determination of prognosis, evaluation of the response to therapies, and monitoring the advancement of pathologies [6,11].

Volatile organic compounds (VOCs) are among the most relevant metabolites produced by the organism. These types of compounds are defined as “… any organic compound having an initial boiling point less than or equal to 250 °C measured at a standard atmospheric pressure of 101.3 kPa” [9], i.e., they are organic compounds that present a volatile state at around room temperature (20–25 °C) [12,13]. Due to their idiosyncrasies, they can interact with organic tissues and be emitted from the interior of the organism, where they are synthesized directly from metabolomic processes to the exterior. In this way, they can be extremely helpful and informative in the field of carcinogenic biomarkers [11]. It is worth mentioning that most of these metabolites are not clinically validated for medical purposes; nonetheless, the potential of all the VOCs addressed in hundreds of scientific publications and throughout this review is undeniable and will certainly lead to their certification in the near future.

Verification of the clinical potential associated with this expanding scientific area is intrinsically connected with the technological development that has allowed reproducible measurements to be carried out through the use of highly precise and sensitive analytical instruments such as GC-IMS (gas chromatography–ion mobility spectrometry) [14,15], GC-MS (gas chromatography–mass spectrometry) [16], GCxGC-TOF-MS (gas chromatography–gas chromatography–time-of-flight–mass spectrometry) [17], PTR-MS (proton transfer reaction–mass spectrometry) [18], SIFT-MS (selected ion flow–tube mass spectrometry) [19], and systems of sensors generally called e-noses (electronic noses) [20]. Specific information on the analytical procedures was not addressed in the present work due to the scope, however, it is worth stating that, with the increase and optimization of detection resources, the number of detectable compounds in breath has also increased to more than 3500 VOCs [7,21].

Dozens of analytes are currently identified as biomarkers for several forms of pathologies [22,23]. The present paper reviews the most relevant bibliographic sources aiming to gather the majority of VOCs already identified as putative cancer biomarkers. In addition, it stresses future perspectives about pathologies whose diagnosis can equally be achieved, in the near future, through volatile biomarkers existent in exhaled air. Most of these pathologies are in a very early stage of research but the promising results already achieved, allied to the valuable potential of breath biomarkers, foresees an auspicious future.

It is important to mention that the literature review reveals a known problem with breath analysis and subsequent identification of potential biomarkers: there is currently no consensus for a standardized methodology that eliminates ambient air influencing factors. In fact, Santos et al. (2023) even studied the suitability of three syringe-based containers for the collection and storage of breath samples. The authors concluded that plastic syringes with rubbered plungers seem to be the proper choice for the mentioned purpose [24]. Nonetheless, this route still requires deeper and more numerous studies to assess the real impact of exogenous factors like temperature, humidity, or collection and storage procedures, as is referred to in this work. In the absence of reliable toxicokinetic models for the absorption and elimination of VOCs from ambient air, the subtraction of VOCs from breathed air must be used with additional care, through the assessment of significant variations in VOCs from ambient air [25].

## 2. Carcinogenic Biomarkers from Breath

Endogenous VOCs identified in exhaled air may arise from metabolic activity in lung tissue and airways or have a systemic origin (produced in any part of the body, including other organs and tissues). However, when produced systemically, these VOCs are captured and distributed in the bloodstream. Thus, the gas exchange of compounds between alveoli and capillaries allows the excretion of exhaled compounds in the form of air, which is exhaled together with respiratory droplets and atmospheric gases. Therefore, the importance of exhaled endogenous VOCs as the main source of biomarkers with clear associations with metabolic status is highlighted.

It is important to highlight that VOCs related to certain diseases may result from metabolic processes that occur, for example, inside a tumour cell and in the surrounding tissues that “react” to the presence of cancer. Lipid peroxidation of polyunsaturated fatty acids, for example, is a biological mechanism that leads to the production of saturated hydrocarbons. Known to be formed in different proportions through chain reactions, ethane and pentane are expelled in greater quantities in situations of mental and/or physical stress during lipid peroxidation. Some methylated hydrocarbons were also identified from breath, although their metabolic pathways are not elucidative enough to confirm their full diagnostic potential [26]. All the compounds and the respective formation processes mentioned here as examples of the origin of the analytes later exhaled in breath are well-known and often detected biomarkers, as will be addressed in due time.

However, to date, only a few VOCs have been officially approved as disease biomarkers. For breath tests, the Food and Drug Administration (FDA) has only approved the following compounds: ethanol (for assessment of blood alcohol content), hydrogen (carbohydrate metabolism), nitric oxide (a biomarker of asthma), carbon monoxide (a biomarker of neonatal jaundice), ^13^CO_2_ (a biomarker of *H. pylori* infection), and branched hydrocarbons (biomarkers of organ transplant rejection) [27]. For exemplification purposes, ethanol is often used by authorities and police figures to assess the alcoholic intoxication levels of drivers [28]. Nitric oxide, in turn, is commonly used in clinical scenarios for the diagnosis of asthma, as mentioned [29]. As can be seen, the world of biomarkers is gaining relevance. Despite the small number of approved biomarkers, several other respiratory metabolites have been referenced as putative breath VOC biomarkers of cancerogenic disease indicators.

**Figure 1:**
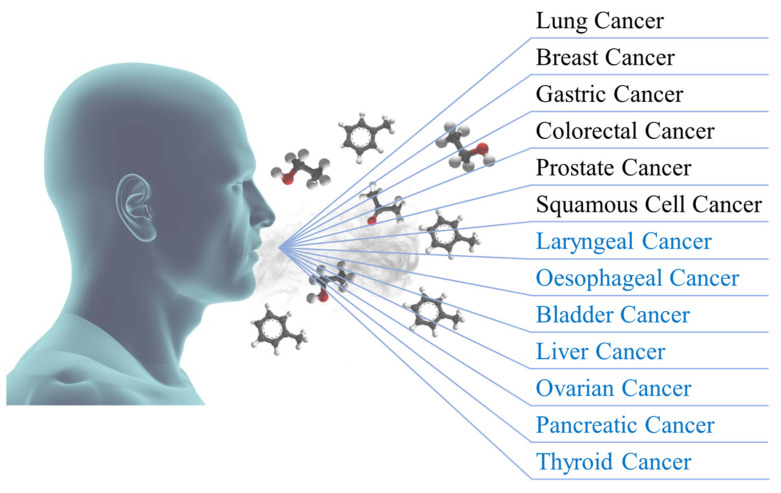
Representation of oncological diseases for which potential breath biomarkers have already been identified (in black) and with future potential biomarkers (in blue).

As mentioned, the scope of the present paper is to review the most relevant scientific sources in order to gather the majority of the volatile organic compounds emitted in exhaled air that are identified as biomarkers for the diagnosis of carcinogenic diseases; namely, breast [30], colorectal [31], gastric [32], lung [33], prostate [34], and squamous cell cancers [35]. Additionally, five other forms of cancer are addressed as future perspectives due to the enormous potential of new VOCs being found in breath for their diagnosis. They are bladder [36], liver [37], ovarian [38], pancreatic [39], and thyroid cancers [40]. Figure 1 schematically represents the pronounced variability of oncological diseases for which potential breath biomarkers have already been identified. For each pathology, a respective table listing, in alphabetical order, the VOCs and the bibliography source for each identified biomarker is also provided. In addition to the VOCs and the respective references, the CAS (Chemical Abstract Service, [41]) and HMDB (Human Metabolome Database, [42]) numbers are also provided for the purposes of chemical and medical identification of the analytes.

### 2.1. Lung Cancer

Lung cancer is the leading and third cause of death among men and women, respectively. Usually caused by behaviours of risk like smoking, it can also be a consequence of long-term exposure to hazardous compounds or environments. In fact, the American Cancer Society estimates that, in the United States of America, a total of 238,340 new cases and 127,070 deaths will occur during the entire year of 2023, which values are extrapolatable for the rest of the world [43]. Due to its high rate of mortality, an accurate and rapid diagnosis is mandatory. However, lung cancer is usually identified through a histological or cytological approach. Both procedures are highly invasive and have some associated risks [44,45,46]. Several biomarkers have been studied and assessed as suitable tools for more rapid and accurate identification of the pathology. Some have even already been validated, and others have presented very promising results [47,48].

In vitro cultures of lung cells were prepared and analysed by Sponring et al. (2010). For that, a thermo-desorption–gas chromatography–mass spectrometry (TD-GC-MS) device was used to separate the analytes present in 200 mL samples of headspace emitted by the cultures. Six specific VOCs were seen to be specifically produced and emitted by the lung cancer cells, in this way, these analytes represent potential biomarkers of the disease [49]. To detect the 18 analytes described as potential lung cancer biomarkers, Chatterjee et al. (2013) developed an electronic nose based on conductive polymer nanocomposite quantum resistive sensors. By applying principal component analysis (PCA) to the collected data, the authors were able to discriminate all the analytes with a total explained variance of 98.34% (PC1—65.11%, PC2—23.69%, PC3—9.54%) [50]. A colourimetric sensor array was developed and applied by Hou et al. (2013) to identify and quantify analytes known for their suitability for the role of lung cancer biomarkers. The authors focused their study on four specific analytes, hexanal, isoprene, p-xylene, and styrene. The device and protocol developed in the study enabled the authors to quantify the mentioned analytes with limits of detection ranging from 50 ppb_v_ to 500 ppb_v_ [51].

The suitability of nanoparticles with magnetic properties to detect several aldehydes in breath samples of lung cancer patients was assessed by Xu et al. (2014). The target VOCs were nonanal, octanal, heptanal, hexanal, pentanal, and butanal, and the authors were able of quantifying them for limits of detection ranging from 2.9 to 21.5 nmolL^−1^ [52]. Gregis et al. (2018) developed an analytical device around a metal oxide-based gas sensor to detect and quantify analytes in the exhaled breath that can correspond to potential biomarkers for lung cancer diagnosis. The device enabled the authors to successfully assess the parameters of four analytes, o-xylene, cyclohexane, propanol, and toluene. The limits of detection for these four compounds were established at around 5, 112, 21, and 24 ppb_v_, respectively [53]. Saalberg et al. (2017) were able to detect six specific VOCs that are widely assessed as possible lung cancer biomarkers. For that, the authors developed a sensor based on photoacoustic spectroscopy with an optical-parametric oscillator as the radiation source. The analytes hexanal, styrene, ethylbenzene, isoprene, propanol, and 2-butanone were identified with detection limits of 15.4, 141.6, 8.6, 36.6, 8.4, and 5.7 ppb_v_ [54].

An ion mobility spectrometer was used to analyse exhaled breath samples of 19 lung cancer patients. The samples were collected through the working channel of a flexible bronchoscope inserted into the patient. The authors were able to detect two analytes whose concentration is significantly higher and three whose concentration is considerably lower in the breath of lung cancer patients. Unfortunately, no information on the identification of the compounds is provided [55]. Mazzone et al. (2012), aiming to describe the breath signature of lung cancer, analysed the exhaled breath of 92 lung cancer patients and 137 healthy controls with a colourimetric sensor array. Even without providing information on the identified VOCs, the authors used the breath signatures of both groups to develop prediction models that were posteriorly validated considering the colour changes of the sensor. These models enabled the authors to differentiate and classify the analysed groups with accuracy levels ranging from 82.5% to 89.0% [56]. A GC-MS device was used by Santonico et al. (2012) to analyse breath samples of healthy individuals and lung cancer patients. The breath collection was performed through an endoscopic probe. Authors could use the respective breath signatures to successfully classify the breath samples in 75% of the cases [57].

The relationship between breath composition and the disease stage was studied by Schmekel et al. (2014) For that, 10 healthy individuals and 22 lung cancer patients were selected as volunteers for breath sample collection and analysis. The analyte separation was performed with an electronic nose (e-nose) and the authors were able to successfully distinguish between the two considered groups. The working principle of the e-nose used by these authors was based on an array of independent sensors, specifically: 10 metal-oxide-semiconductor field effect transistors and 12 metal-oxide semiconductor sensors, capable of detecting alkanes and nucleophilic compounds, respectively [58]. Nine volatile organic compounds were assessed by Li et al. (2015) regarding their suitability for the role of lung cancer biomarkers. For that, 85 healthy individuals, 34 benign pulmonary nodules patients, and 85 lung cancer patients were considered for breath analysis by Fourier transform–ion cyclotron–mass spectrometry (FT-ICR-MS). By considering the respective breath signatures, authors could successfully distinguish lung cancer patients from non-smokers (97% accuracy), smokers (95% accuracy), and benign pulmonary nodule patients (89% accuracy) [59].

To validate the suitability of breath biomarkers as a diagnostic tool for lung cancer in two independent cohorts, Phillips et al. (2015) developed a dual study. The first considered cohort was composed of 35 healthy individuals, 82 healthy smokers, 84 high-risk symptomatic patients, and 100 lung cancer patients. The second cohort, in its turn, included 19 healthy individuals, 70 healthy smokers, 51 high-risk symptomatic patients, and 75 lung cancer patients. By considering the respective breath prints, the authors could discriminate the lung cancer patient group in the first cohort with sensitivity and specificity levels of 68.0% and 68.4%. The sensitivity and specificity levels of lung cancer patient group discrimination in the second cohort were 70.1% and 68.0%, respectively [60]. The control group (89 individuals) and lung cancer patient group (81 individuals) were considered in a study developed by Callol-Sanchez et al. (2017). Here, the collected samples of breath were analysed with a GC-MS device, and special emphasis was given to nonanoic acid, propanoic acid, nonanal, octanal, heptanal, and hexanal. All of these VOCs have been studied as high-potential analytes for lung cancer diagnosis. In fact, the authors were able to prove that nonanoic acid, for example, is 2.5 times more likely to be present in the breath of lung cancer patients than in healthy breath samples [61].

A proton transfer reaction–mass spectrometry (PTR-MS) device was used to separate the analytes of exhaled breath samples collected from lung cancer patients (30 subjects) and healthy individuals (30 subjects). By considering the respective breath signatures, Sun et al. (2019) were able to discriminate the groups with promising interval levels of accuracy (74–99%), sensitivity (60–100%), and specificity (73–97%) [62]. Exhaled breath samples of healthy volunteers (12 subjects) and lung cancer patients (32 subjects) were collected into inert Tedlar bags and analysed with a GC-ToF-MS device by Saidi et al. (2020). The authors were able to detect 30 VOCs with potential for the role of lung cancer biomarkers and, additionally, four of those stand out from the others due to their even higher discriminant capacity. Unfortunately, the authors did not provide the identification of these four VOCs [63].

Nonanal, octanal, hexanal, and pentanal were identified as discriminant analytes for lung cancer by Fuchs et al. (2010). The concentration levels of these analytes were considerably higher in the breath of lung cancer patients than in healthy individuals’ breath; hence, the four VOCs were considered to be potential biomarkers for lung cancer. To achieve such results, the authors performed analyses of the breath of 12 lung cancer patients and 12 healthy subjects. The identification and quantification of the analytes was made possible by a GC-MS device [64]. Several aldehydes were also assessed as potential lung cancer biomarkers by Poli et al. (2010). These seven analytes (propanal, butanal, pentanal, hexanal, heptanal, and octanal) were identified and quantified in breath samples collected from lung cancer patients (40 subjects) and healthy individuals (38 subjects), with a solid-phase microextraction–gas chromatography–mass spectrometry (SPME-GC-MS) device [65]. Exhaled breath samples of 23 lung cancer patients and 30 healthy volunteers were collected into 1 L Tedlar bags and posteriorly analysed with a GC-ToF-MS device. Among all the detected analytes, Rudnicka et al. (2011) were able to identify five specific VOCs (isopropyl alcohol, ethylbenzene, 2-propenal, carbon disulphide, and propane) that enable the best discrimination between both groups. Consequently, the mentioned analytes have a high potential for the role of lung cancer biomarkers [66].

A total of 12 VOCs were identified by Buszewski et al. (2012), with a GC-ToF-MS device and with limits of detection ranging from 0.31 ppb_v_ to 0.75 ppb_v_. A cohort of 73 individuals (29 lung cancer patients and 44 healthy subjects) was gathered for the breath analyses. The authors conclude that all 12 analytes show an increase in their concentration levels in the exhaled breath of lung cancer patients [67]. In a parallel study, Buszewski et al. (2012) used an SPME-GC-MS device to analyse the headspace emitted by tissue samples collected from oncologic patients. The authors conclude that eight VOCs (2-pentanone, 2-butanone, 2-propanol, 1-propanol, dimethyl sulphide, carbon disulphide, acetone, and ethanol) presented higher levels of concentration when emitted from carcinogenic tissues when compared with normal tissues [68].

A Fourier transform–ion cyclotron resonance–mass spectrometry (FT-ICR-MS) device was used by Fu et al. (2013) to assess the breath analytes of healthy volunteers (88 subjects) and lung cancer patients (97 subjects). From the dozens of detected analytes, four VOCs exhibited a similar change of behaviour between healthy volunteers and lung cancer patients. The concentration levels of 4-hydroxy-hexenal, 3-hydroxy-2-butanone, 2-butanone, and 2-hydroxy-acetaldehyde were consistently higher in the breath of lung cancer scenarios [69]. Ma et al. (2014) used a solid-phase microextraction–gas chromatography–gas chromatography (SPME-GC-GC) device to analyse the exhaled breath samples of 38 individuals (25 healthy volunteers and 13 lung cancer patients). Among all the detected analytes, five specific VOCs (propanol, pentane, methanol, isoprene, and acetone) were identified as potential biomarkers. In fact, these analytes present higher concentration levels in the breath of lung cancer patients than in healthy volunteers’ breath [70].

An ion mobility spectrometry (IMS) device was the analytical technique selected by Handa et al. (2014) to analyse exhaled breath samples collected from 39 healthy volunteers and 50 lung cancer patients. Among the 115 detected VOCs, a single analyte (dodecane) enabled the authors to differentiate both groups with sensitivity and specificity levels up to 76% and 100%, respectively [71]. Eight volatile organic compounds were identified by Ligor et al. (2015) as potential lung cancer biomarkers due to their discriminating capacity between oncologic and healthy cases. A cohort of 484 volunteers (361 healthy subjects and 123 lung cancer patients) was used for breath analyses with an SPME-GC-MS device. The mentioned VOCs enabled the authors to differentiate both groups with accuracy, sensitivity, and specificity levels of 65.0%, 63.5%, and 72.4%, respectively [72]. Octanal was identified as a potential biomarker for lung cancer detection by Jouyban et al. (2017). For that, the authors collected 1000 mL of exhaled breath into a homemade extraction device. The separated VOCs were condensed in 0.5 mL of acetone and posteriorly analysed with a GC-MS device. The limits of detection and quantification for the octanal were 0.008 ng/mL and 0.026 ng/mL, respectively [73].

As reviewed, the study of exhaled breath biomarkers for the diagnosis of lung cancer, in the form of volatile organic compounds is very complete and deeply assessed. There are several VOCs already validated and certified as biomarkers; nevertheless, other analytes require further investigation to assess their suitability. Table 1 summarizes the main VOCs addressed in the reviewed works, including their respective references, and CAS and HMDB numbers for the purposes of chemical and medical identification.

### 2.2. Breast Cancer

Breast cancer is among the most common types of carcinomas in women and, additionally, leads to thousands of deaths every year. In fact, the American Cancer Society and the Cancer Research Institute of the United Kingdom estimate that a total of 297,790 and 55,920 new cases will be diagnosed during the entire year of 2023, respectively, for the United States of America and the United Kingdom. Regarding the deaths, a total of 43,700 and 11,499 are, respectively, expected during 2023—impressive numbers [74,75].

Contemporary unhealthy lifestyles like poor nutrition or lack of exercise, and changes in reproductive behaviour are some of the main risk factors for the rise of breast cancer incidence [76,77]. In addition to their origins, the current methods for breast cancer screening and diagnosis are often aggressive and painful due to the necessity of invasive procedures. This fact leads to a later diagnosis of the pathology and, consequently, to more dangerous comorbidities [78,79]. Several studies have been developed aiming at the identification of possible biomarkers emitted mainly in the breath, but also in the urine, of the patients, that allow fast, non-invasive, and painless screening for breast cancer [80,81,82,83].

Aiming to differentiate between sub-types of breast cancer tumours, Barash et al. (2015) analysed the breath of 276 women with different types of lesions. As in some of the previously addressed papers, a GC-MS device was the analytical technique selected to perform the analyses. A total of 23 analytes of interest were detected and 13 were accurately identified [84]. To study the fingerprint patterns of VOCs emitted from four different types of breast cancer cells, Lavra et al. (2015) also used a GC-MS device. From the in vitro cultures of cells, the authors were able to successfully identify 13 specific VOCs with elevated potential for being breast cancer biomarkers [85]. In vitro cultures of breast cancer cells were also prepared and studied by Arshad et al. (2014). By using a Fourier-transform infrared (FTIR) spectroscopy device, the authors were able to remark methanol as a clear biomarker for breast cancer [86]. Ethyl acetate, 2-methyl butanoate, ethyl propanoate, 3-methyl-3-buten-1-ol, 2-heptanone, and 2-pentanone were identified in the headspace of in vitro cell cultures, by Silva et al. (2017) as VOCs with high potentiality for being biomarkers of breast cancer [87].

To assess the suitability of VOCs as biomarkers for breast cancer diagnosis, Phillips et al. (2014) analysed the exhaled breath of 244 women divided into two groups, healthy individuals and women with abnormal screening mammograms (breast cancer). Through the detection of breath signatures, the authors were able to differentiate both groups with accuracy, specificity, and sensitivity levels of 79, 70 and 81.8%, respectively. Screening mammograms were used to check the results [88]. Ji et al. (2014), with a similar goal, analysed the exhaled breath of 63 volunteers (24 healthy individuals, 22 breast cancer patients, and 17 benign breast tumour patients). The breath signatures collected from the three groups enabled a successful differentiation among them. The distinction between healthy women and breast cancer patients was achieved with sensitivity and specificity levels of 68.2 and 91.7%, respectively. The sensitivity and specificity values for the differentiation between benign and malignant breast cancer were 91.7 and 95.8%, respectively [89]. GC-MS was used by Phillips et al. (2017) to analyse the exhaled breath of 258 women (54 breast cancer patients and 204 healthy volunteers). Even without information on the identified VOCs, 21 analytes were detected as potential biomarkers. The discrimination of breast cancer patients from healthy women considering these VOCs was achieved with an accuracy level of 77% [90]. In a more recent study, Phillips et al. (2018) analysed the exhaled breath of 178 women (54 breast cancer patients and 124 healthy volunteers) with two analytical techniques, a GC-MS device and a gas chromatography–surface acoustic wave detection (GC-SAW) device, for comparison purposes. Again, no information on the identification of VOCs is provided; however, the breath signatures detected by GC-MS enabled the differentiation of both groups with 90% accuracy. The results of the GC-SAW measurements enabled the authors to distinguish between both groups with 86% accuracy [91]. Finally, Yang et al. (2021) were able to differentiate both groups of healthy individuals (*n* = 88) and breast cancer patients (*n* = 351) through their respective VOCs signatures in breath, with accuracy, sensitivity, and specificity levels of 91, 86, and 97%, respectively [92].

To identify breath signatures for breast cancer with an electronic nose, León-Martínez et al. (2020) analysed the breath samples of 443 women (181 healthy volunteers and 262 breast cancer patients). The commercial e-nose used by the authors bases its working principle on the variation of electrical resistance of 32 polymer-based sensors once exposed to the target metabolites. Unfortunately, the authors do not provide information on the identified analytes, but they were able to use the VOCs’ patterns in the breath to differentiate between healthy women and breast cancer patients in 98% of the cases [93]. In a study developed by the same research group, Rodríguez-Aguilar et al. (2021) were able to distinguish breast cancer patients from lung cancer patients and chronic obstructive pulmonary disease (COPD) patients through the breath signatures collected from each group. The measurement data were processed by principal component analysis (PCA). The differentiation between lung and breast cancer patients was achieved with a total explained variance of 98.6% (PC1—80.1%, PC2—17.2%, PC3—1.3%). Regarding the differentiation between COPD and breast cancer patients, the resulting total explained variance was 99.2% (PC1—96.3%, PC2—2.0%, PC3—0.9%) [94].

Phillips et al. (2010) analysed the exhaled breath of 258 women (54 breast cancer patients and 204 healthy volunteers) with a GC-MS device. The authors employed a portable breath collection apparatus to sample 1 L of alveolar breath, during 2 min of normal breathing through a disposable inert mouthpiece. From the detected analytes, authors isolated 10 with a higher potential for being biomarkers and, for their identification, 26 possibilities were gathered [95]. A cohort of 20 individuals (10 healthy individuals and 10 breast cancer patients) was also analysed with GC-MS by Mandy et al. (2012). Samples of 1 L of alveolar breath were collected and four VOCs, decene, naphthalene, caryophyllene, trichloroethylene, and 3-methyl hexane, were identified due to their high potential for being breast cancer biomarkers [96]. Xu et al. (2013) developed a chip with a highly sensitive single nanowire array to detect and identify VOCs in breath potentially related to breast cancer. Four specific VOCs, heptanal, 2-propanol, isopropyl myristate, and acetophenone, were successfully identified by the authors as potential biomarkers. The detection limits ranged from 798 ppb_v_ to 129.5 ppm_v_ [97]. P-xylene, cyclohexanol, 2,4-dimethyl-benzaldehyde and 2-ethyl-1-hexanol were identified by Huang et al. (2016) as potential biomarkers for discriminating breast cancer cells from healthy mammary cells. In vitro cultures of both healthy and cancerous cells were prepared, and the emitted headspace was analysed with a solid-phase microextraction–gas chromatography–mass spectrometry (SPME-GC-MS) device [98]. Fifteen VOCs were identified by Zhang et al. (2020) as potential biomarkers for breast cancer diagnosis. For that, the authors analysed the exhaled breath of 203 volunteers with an SPME-GC-MS device [99]. As the reviewed studies prove, the field of breast cancer biomarkers, and specifically, VOCs as biomarkers, is a developing world that already has very promising and paradigm-changing results. Table 2 summarizes the main VOCs addressed in the reviewed works including their respective references, and CAS and HMDB numbers for the purposes of chemical and medical identification.

### 2.3. Gastric Cancer

Most of the techniques currently used for diagnosing gastric cancer involve invasive painful procedures. For example, a digestive endoscopy with biopsy for posterior diagnosis through histopathological analysis requires the introduction of medical devices for the collection of tissue samples. The development of non-invasive but accurate techniques for gastric cancer diagnosis would enable a faster reaction and treatment of the disease [101,102,103]. The rapidness and effectiveness of the diagnostic are particularly important since the number of new cases is impressive. In fact, just for the United States of America, the American Cancer Society estimates that, during 2023, 26,500 new cases and 11,130 deaths will occur of gastric cancer [104].

Some studies have been developed to identify possible biomarkers for the diagnosis of gastric cancer. Salivary VOCs, for example, were studied for this purpose by Bel’skaya et al. (2020). The identified VOCs (ethanol, 2-propanol, acetone, and acetaldehyde) enabled the authors to differentiate between healthy individuals and gastric cancer patients with specificity and sensitivity levels of 90.9% and 95.7%, respectively [105]. Regarding the exhaled breath, an electronic nose (e-nose) was used by Schuermans et al. (2018) to analyse a cohort of 44 individuals (28 healthy volunteers and 16 gastric cancer patients). This device bases its working principle on the heating and cooling of three micro-hotplate metal-oxide sensors that, once exposed to the metabolites exhaled in breath, alter their conductivity, leading to the formation of VOC-specific patterns. The identification of the VOC breath signature for each group enabled the authors to differentiate them with accuracy, sensitivity, and selectivity levels of 75%, 81%, and 71%, respectively. Unfortunately, information about the detected VOCs is not provided [106]. Zhang et al. (2014), in their turn, studied the analytes emitted by in vitro cultures of gastric cancer cells. The headspace emitted by the cultures of cells was analysed by gas chromatography–mass spectrometry (GC-MS). Eight volatile organic compounds were successfully identified as being produced solely by the cancer cells and, hence, are potential biomarkers for gastric cancer diagnostics [107].

Xu et al. (2013) developed a breath test whose aim is to differentiate benign gastric cancer from gastric cancer by examining the breath signatures of both groups. For that, the exhaled breath of a total of 130 patients (37 gastric cancer patients and 93 patients with non-oncologic gastric pathologies) was analysed. The results obtained enabled the authors to identify five VOCs with high suitability for being biomarkers. These five VOCs allowed differentiation between gastric cancer and less severe gastric pathologies with a sensitivity and specificity of 84% and 87%, respectively [108]. Furfural, 2-propene-nitrile, and 2-butoxy-ethanol were also identified as gastric cancer biomarkers, among other VOCs, by Amal et al. (2016). In addition, four other VOCs were also remarked on as being potential biomarkers (4-methyl octane, 2-butanone, 1,2,3-trimethylbenzene, and α-methyl-styrene). The identification of these analytes from the exhaled breath analysis of 484 patients (99 gastric cancer patients and 385 healthy volunteers) enabled the authors to differentiate between both groups with levels of accuracy, sensitivity, and specificity of 92%, 73%, and 98% [109].

Nine analytes were identified in the exhaled breath of 43 volunteers (17 healthy subjects and 26 gastric cancer patients), by Jung et al. (2021). A proton-transfer-reaction time-of-flight mass spectrometry (PTR-ToF-MS) device was the analytical technique selected for the analysis. The identification of the mentioned VOCs as potential gastric cancer biomarkers was achieved with an accuracy level of 82% [110]. A thermal desorption–single-photon ionization–mass spectrometry (TD-SPI-MS) device was used by Hong et al. (2021) to analyse the exhaled breath of a large cohort (174 volunteers: 78 healthy subjects and 96 gastric cancer patients). Among all the detected analytes, the authors were able to identify seven specific VOCs with the potentiality of being gastric cancer biomarkers. By using these seven VOCs in a single pattern, the differentiation of both groups was achieved with an accuracy level of 96.2% [111]. Chen et al. (2016), in their turn, were able to identify 14 volatile organic compounds as potential biomarkers for gastric cancer diagnosis. A surface-enhanced Raman scattering (SERS) sensor was the selected procedure to analyse and differentiate between healthy volunteers and gastric cancer patients. The differentiation was achieved by the authors with sensitivity and specificity values of 83% and 92% [112]. As reviewed, there are several promising results in the field of gastric cancer biomarkers. Further scientific studies must be developed to make this possibility a reality. Table 3 summarizes the main VOCs addressed in the reviewed works including their respective references, and CAS and HMDB numbers for the purposes of chemical and medical identification.

### 2.4. Colorectal Cancer

Colorectal cancer (CRC) is one of the cancers with a higher incidence and mortality. In fact, just in the United States of America, 106,970 new cases and 46,050 deaths are expected during 2023 [113]. Since it can be a very silent pathology, CRC is usually detected only in the late stages of development, leading to very low rates of cure [114,115]. New, precise and, more importantly, rapid diagnostic tools are mandatory. The application of VOCs as an option for an accurate diagnosis of CRC has been deeply studied and this has already provided substantial results [116,117].

Altomare et al. (2013) collected breath samples into 1 L inert Tedlar bags, from 41 healthy individuals and 37 colorectal cancer patients. The samples were analysed with a GC-MS device. Among all the identified compounds, the authors were able to build a pattern composed of 15 VOCs that distinguishes both groups with sensitivity and specificity levels of 86% and 83%, respectively [118]. In follow-up work, the same research group analysed the breath of 103 volunteers (55 healthy individuals and 48 CRC patients) with a thermal-desorption–gas chromatography–mass spectrometry (TD-GC-MS) device. The authors were able to identify 12 of the 15 VOCs detected in the previous work. In addition, the differentiation between both considered groups, based on their breath signatures, was achieved with accuracy, sensitivity, and specificity levels of 98.6%, 100.0%, and 97.9%, respectively [119]. Finally, in a third study, Altomare et al. (2020) analysed the exhaled breath of 83 CRC patients and 90 healthy subjects. Among all the detected analytes, 14 VOCs were identified as high-potential biomarkers for the diagnosis of CRC [120].

Nine VOCs were identified by Wang et al. (2014) as potential biomarkers for the diagnosis of colorectal cancer. To achieve this, the exhaled breath of 40 individuals (20 healthy subjects and 20 CRC patients) was collected and analysed with an SPME-GC-MS apparatus [31]. A total of 122 healthy individuals and 87 CRC patients were considered by Amal et al. (2016) for breath analysis with a GC-MS. The authors concluded that two specific VOCs, ethyl acetate and acetone, increase their concentration levels (in the ppb_v_ range) in the breath of CRC patients. On the other hand, 4-methyl octane and ethanol decrease their concentration (in the ppb_v_ range) in these cases. In conclusion, the referred to VOCs represent potential biomarkers for the diagnosis of CRC [121]. Even without identifying the VOCs, Keulen et al. (2020), could differentiate healthy individuals (68 subjects) from CRC patients (42 subjects). This discrimination was based on the breath signatures of both groups, and it was achieved with sensitivity and specificity levels of 83% and 60%, respectively [122]. Four acids (butanoic, pentanoic, dodecanoic and octanoic acids) and seven other VOCs were recently identified by Vietro et al. (2020) as potential CRC biomarkers. For that, exhaled breath samples were analysed with a GC-MS apparatus [123]. Considering the reviewed works, it is possible to state that volatile organic compounds play a crucial role in contemporary procedures for CRC diagnosis. Table 4 summarizes the main VOCs addressed in the reviewed works including their respective references, and CAS and HMDB numbers for the purposes of chemical and medical identification.

### 2.5. Prostate Cancer

As with other urogenital system cancers, prostate cancer has a very challenging diagnosis, and, in most cases, identification of the pathology is only determined in the late stages when the cure probabilities are diminished. An accurate methodology for diagnosing prostate cancer commonly involves blood analysis and, in most cases, biopsy-based procedures that are deeply invasive and present some risks to the patient. In this way, this pathology, which leads to thousands of deaths among men every year (12,000 and 34,700 deaths, respectively, are expected in the United Kingdom and the United States of America during 2023, [124,125]), requires more rapid and more accurate diagnostic methodologies to tackle the current challenges [126,127]. A lot of work has been developed around urinary VOCs, however, breath biomarkers for prostate cancer remain a scientific area filled with opportunities [128,129].

Waltman et al. (2018) have recently addressed the suitability of an electronic nose device to identify patterns of VOCs in the exhaled breath of both prostate cancer patients (32 individuals) and healthy volunteers (53 individuals). The working principle of this commercially available e-nose consists of the creation of compound-specific patterns by hotplate metal-oxide sensors, whose conductivity varies once exposed to the target analytes. This variant, as mentioned, is directly related to the detected analyte, leading to a unique fingerprint of the studied matrix. Unfortunately, information about the detected VOCs is not provided but the authors were able to use breath signatures to distinguish both groups with accuracy, sensitivity, and specificity levels of 75%, 84%, and 70%, respectively [130]. In even more recent work, Maiti et al. (2021) analysed breath samples collected from healthy subjects (19 individuals) and prostate cancer patients (28 individuals) in the search for potential biomarkers. Six VOCs, methyl butyrate, ethyl butyrate, acetaldehyde, ethyl vinyl ketone, propyl propionate and acetic anhydride, were identified by the authors as being of extreme relevance for the diagnosis of prostate cancer through breath biomarkers (Table 5) [131]. Table 5 summarizes the main VOCs addressed in the reviewed works including their respective references, and CAS and HMDB numbers for the purposes of chemical and medical identification.

The reviewed works prove the necessity of additional studies addressing the issue but the already achieved results are very promising. Nonetheless, it is evident that the field of breath biomarkers for prostate cancer diagnosis has a long way to go. As can be seen, few studies have been developed regarding these breath biomarkers, perhaps due to the complexity of such studies. It is important to state that the reviewed works prove the auspicious future of this field and we could be seeing the certification and utilization of breath biomarkers in the medical field in the near future; steps that would be deeply important because they would help reduce the invasiveness of the current procedures, reduce the time required for the diagnosis of these diseases, expediting treatment and, consequently, diminish mortality rates.

### 2.6. Squamous Cell Cancer

Commonly known as head and neck cancer (HNC), squamous cell carcinoma (SCC) encompases several types of cancer. Tumours whose development occurs in any head structure, like craniofacial bones, mucosa of the oral cavity, soft tissues, or even skin, are usually defined as HNC. The treatment of HNCs is especially difficult due to their location. Any kind of treatment deeply interferes with very important structures involved with necessary-to-life activities, like eating or even breathing. In this way, a rapid and accurate diagnosis of the tumour type and location is crucial to avoid more severe and invasive treatments [132,133,134]. The physicians and researchers working with HNC patients are in constant search of new methodologies that enable a faster and more accurate diagnosis of the pathology. The identification of specific VOCs as disease biomarkers has been interpreted as one of the solutions with higher potential [135,136,137].

To assess the suitability of patterns of VOCs as potential biomarkers for HNC diagnosis, Leunis et al. (2013) analysed exhaled breath samples with an electronic nose device. This device employs a total of 12 metal-oxide sensors specifically developed for the detection of four types of metabolites (CH4, CO, NOx, and Pt). Samples of the exhaled breath from 59 individuals (23 healthy subjects and 36 HNC patients) were collected into 5 L Tedlar bags and posteriorly analysed with the e-nose. The authors claimed that the differentiation between both groups was achieved with a specificity and sensitivity of 80% and 90% [138]. In a three-dimensional study, Goor et al. (2016) evaluated the breath of patients with three distinct pathologies: colon (28 patients), bladder (40 patients), and head and neck (100 patients) cancers. Even without information on the detected VOCs, the authors were able to use the detected patterns to differentiate HNC patients from colon and bladder cancer patients with accuracy levels of 81% and 84%, respectively [139]. In a more recent study, the same research group analysed the exhaled breath of HNC patients after treatment procedures. A cohort of 40 volunteers (20 patients with tumour recurrence and 20 patients without evidence of a tumour) was analysed with an e-nose device. The e-nose specifically used in this study consists of three hotplate metal-oxide sensors that are continuously heated and cooled down and that, once exposed to the target analytes, vary their conductivity. This process leads to the formation of VOC-specific patterns that act as a fingerprint of the samples under analysis. In this situation, the pattern of VOCs was not identified; however, the authors were able to use the patients’ breath signatures to discriminate between the two groups with an accuracy level of 83% [140]. Hartwig et al. (2017), in their turn, used a GC-MS device to analyse the exhaled breath of 14 volunteers (4 healthy controls and 10 HNC patients). Among the 125 analytes detected in the exhaled breath, the authors found eight VOCs with high potential as HNC biomarkers. Three of these eight compounds disappeared after surgery to remove the tumour [141]. The addressed results prove the potentiality of breath signatures to rapidly, accurately, and noninvasively diagnose HNC.

Some scientific research has been carried out regarding urine-emitted VOCs as biomarkers for several types of cancer, as that completed by Opitz et al. (2018) [142]. Regarding the VOCs emitted in the exhaled breath, interesting studies have been conducted. A cohort of 47 subjects (34 healthy individuals and 13 neck cancer patients) was considered for a study developed by Zhou et al. (2017). The use of a proton transfer reaction–mass spectrometry (PTR-MS) enabled the authors to detect four characteristic analytes with high potential for HNC diagnostic. The complete identification of these four analytes was not achieved but the authors were able to reduce the list of possibilities to just 22 VOCs [143]. Hakim et al. (2011), in a previous study, addressed the HNC issue. From the analyses of the breath samples of 87 individuals with a nanoscale artificial nose (NA-NOSE), the authors built the patterns of VOCs for both healthy people and HNC patients; with a GC-MS device, it was possible to identify the detected VOCs. Among all the detected volatile organic compounds, 12 were identified as high-potential biomarkers for diagnosing and distinguishing HNC from other pathologies [144]. A GC-IMS apparatus was used by Taware et al. (2018) to identify four volatile organic compounds whose behaviour is directly related to the diagnosis of HNC. From the breath analyses of 59 individuals (27 healthy individuals and 32 HNC patients), 48 analytes were detected [145]. Hydrogen cyanide was the VOC identified by Chandran et al. (2019) as a clear biomarker for HNC diagnosis. The differentiation of HNC patients and healthy groups through this analyte was achieved with an accuracy level of 95% [146]. Gruber et al. (2014), in their turn, identified three volatile organic compounds, undecane, ethanol, and 2-propenonitrile, as HNC-differentiating markers [147].

All the reviewed works prove that the role of VOCs as HNC biomarkers is of extreme relevance; however, further work must be carried out. One can state that the final aim of this field is to achieve the certification of breath biomarkers for HNC diagnosis and, consequently, their implementation in real clinical scenarios. To do so, additional techniques with better separation and detection capacities must be tested. Techniques like ion mobility spectrometry (IMS), whose limits of detection can achieve levels as low as ppb_v_ and even ppt_v_, can be a suitable option to detect eventual metabolites not identified yet as relevant for this field [15]. In addition, newer collection and storage procedures based on new devices must be developed and tested in order to ensure the repeatability of the data and the relevancy of the results [24,148]. Beyond all the current challenges, the future of breath biomarkers for HNC diagnosis is auspicious and should not be overlooked.

As mentioned, HNC includes diverse types of oncologic diseases. Here, two of the most common HNCs are addressed: laryngeal and oesophageal cancer. The scientific studies aimed directly at these two pathologies are reviewed.

#### 2.6.1. Laryngeal Cancer

Laryngeal cancer affects mainly people exposed to high-risk behaviours like consuming alcohol or smoking. With around one million cases every year, and tending to increase, new, rapid, and accurate diagnostic techniques and procedures are desired. The identification of biomarkers among the VOCs exhaled in patients’ breath has gained relevance recently due to its high diagnostic accuracy [149,150,151].

Ethanol and 2-butanone were identified as clear biomarkers for laryngeal cancer by García et al. (2013). Their concentration levels are expressively distinct in the exhaled breath of healthy individuals and oncologic patients. Two other VOCs, 2,3-butanediol and 9-tetradecen-1-ol, were also considered of great potential for diagnosing laryngeal cancer but further studies are required. To achieve such results, the authors analysed the breath samples of 31 volunteers (11 cancer patients and 20 healthy subjects) with a solid-phase microextraction–gas chromatography–mass spectrometry (SPME-GC-MS) device [152]. An electronic nose was used by Shoffel-Havauk et al. (2015) to analyse the breath samples of both healthy people and laryngeal cancer patients. Four volatile organic acids were identified as potential biomarkers for laryngeal cancer diagnosis, namely, heptanoic acid, hexanoic acid, pentanoic acid, and butyric acid; however, the authors claim that further studies are required to ensure that these analytes are directly originated or related to the pathology [153].

In summary, the field of breath biomarkers for laryngeal cancer diagnosis is promising; however, there are still some major challenges to overcome. The poor sensitivity/specificity of the analytical techniques used to perform the analysis often prevents the detection of metabolites present at trace levels of concentration. In addition, some of the current procedures for breath collection and storage are not completely safe and often lead to the contamination and degradation of the samples. More appropriate techniques must be tested for the study of breath samples, and newer collection procedures should be developed and implemented. These new approaches and studies must have one single goal in mind, the certification of breath biomarkers for medical applications.

#### 2.6.2. Oesophageal Cancer

A sub-type of squamous cell carcinoma, oesophageal cancer affects thousands of people worldwide. The increasing number of risk factors and activities, like smoking, is leading to an equal increase in oesophageal cancer incidence. The identification of biomarkers in the exhaled breath of patients has been deeply studied regarding its potential for a rapid, non-invasive, and accurate diagnosis [148,154,155].

Aiming to differentiate oesophageal cancer patients (29 patients) from healthy individuals (57 healthy subjects) through their VOC signatures, Zou et al. (2016) collected and analysed exhaled breath samples with a proton transfer reaction–mass spectrometry (PTR-MS) device. The differentiation between both groups was achieved with sensitivity and specificity levels of 86.2% and 89.5%, respectively [156]. In a more recent study, Markar et al. (2018) gathered a large cohort of 325 volunteers (163 oesophageal cancer patients and 172 healthy individuals). Steel breath bags with 500 mL capacity were used for sampling the exhaled breath of all the participants. A SIFT-MS device was used for the analysis procedures. By considering a pattern of five analytes, the authors were able to differentiate both groups with diagnostic accuracy, sensitivity, and specificity levels of 85%, 80%, and 81%, respectively [157]. A total of four VOCs (hexanoic acid, phenol, methyl phenol and ethyl phenol) were identified by Kumar et al. (2013); a selected ion flow tube–mass spectrometry (SIFT-MS) device was used to analyse the exhaled breath of healthy individuals and oesophageal cancer patients. By considering the identified VOCs, the authors were able to differentiate between both groups with an accuracy of 91% [158].

The reviewed papers prove the vast number of studies that have been developed in the field of VOCs as biomarkers for the diagnosis of all types of squamous cell carcinoma. In addition, they also highlight some of the current challenges, namely, the lack of standardized procedures to collect, transport and store the exhaled air samples, preventing their contamination and degradation. One can also state that further studies should be developed around analytical techniques with higher detection capacities and higher sensitivity levels in order to explore eventual biomarkers present at trace levels of concentration.

Table 6 summarizes the main VOCs addressed in the reviewed works including their respective references, and CAS and HMDB numbers for the purposes of chemical and medical identification.

### 2.7. Pathologies with Research Potential

Several pathologies deserved to be addressed in this review; however, in a distinct section. Most of the following pathologies still require deeper scientific knowledge in the area of breath biomarkers and, hence, their identification through the emitted VOCs is still a field requiring deeper exploration. Other diseases are known to have biological-borne biomarkers emitted by the human body; however, this emission does not occur through the breath. Broadly speaking, the health conditions included here deserve special mention due to their currently unexplored potential.

As addressed in the following chapters, several works have been carried out but major challenges must be overcome in order to achieve the final goal for all these fields, the certification of breath biomarkers for clinical diagnosis and medical applications. Some of those challenges are related to the lack of standardized procedures for collecting and storing exhaled samples. This type of sample is easily spoilable and can be effortlessly contaminated, so better procedures are required. In addition, some of the biomarkers are often emitted in the breath at trace levels of concentration, i.e., ppb_v_ or even ppt_v_, which makes their detection extremely difficult through some of the analytical techniques commonly used in this kind of study. Once all the issues have been overcome, and considering the promising results already achieved, one can state that the field of breath biomarkers has an auspicious future and can lead to the modernization of the medical field.

#### 2.7.1. Bladder Cancer

Responsible for thousands of new cases every year (82,290 new cases are expected during 2023 just in the United States of America [159]), bladder cancer has a rate of survival close to 100% if detected in the first stage of development. Considering this fact, a rapid and accurate diagnosis procedure is mandatory to increase the chances of survival [36,160]. Several studies addressing urinary VOCs as potential biomarkers have been developed; however, breath analytes are yet to be explored [161,162].

To differentiate three distinct groups of patients, head and neck, colon, and bladder cancer patients, Goor et al. (2016) analysed exhaled breath samples with an electronic nose (e-nose). The commercial device used by the authors produces a specific pattern of the analysed sample due to the continuous reduction–oxidation reactions of the VOCs on the surfaces of the hotplate metal-oxide sensors assembled in the interior of the apparatus. Considering the VOC signature of each group, the authors were able to differentiate bladder cancer from head and neck cancer patients with sensitivity and specificity levels of 80% and 86%. The differentiation between bladder cancer and colon cancer patients through their respective patterns of VOCs was achieved with a sensitivity and specificity of 88% and 79%, respectively. [139]. A long path is yet to be traversed in this field; however, the potentialities are endless.

#### 2.7.2. Liver Cancer

The survival rate of liver cancer is, frankly, low since most cases are diagnosed in the later stages of development. Even nowadays, the rate of successful treatment is under 20%. Liver cancer is one of the pathologies that require faster and more accurate diagnosis procedures to diminish mortality (nearly 30,000 deaths are expected in the United States of America during 2023) [163,164]. The suitability of VOCs as potential biomarkers for rapidly and accurately diagnosing liver cancer has been assessed in a few studies [165]. Amal et al. (2012) and Bannaga et al. (2021) have, respectively, analysed the VOCs emitted by blood and urinary samples collected from liver cancer patients [166,167]. The potential of breath analytes as biomarkers was assessed by some research groups. Qin et al. (2010), for example, analysed breath samples of 66 volunteers (36 healthy individuals and 30 liver cancer patients) and identified decane, styrene, and 3-hydroxy-2-butanone as VOCs of interest. By using these three VOCs as a pattern, the authors were able to successfully differentiate between both groups with sensitivity and specificity levels of 86.7% and 91.7%, respectively [168]. Miller-Atkins et al. (2020) identified 22 VOCs in the breath of both liver cancer patients and healthy individuals as potential biomarkers. Considering these analytes, the pathological cases were distinguished from healthy ones with accuracy and sensitivity levels of 85%, and 73%, respectively [169], proving the long but promising path that must be explored in the field of liver cancer biomarkers.

#### 2.7.3. Ovarian Cancer

Ovarian cancer is a type of gynaecological cancer with a higher rate of mortality among women and, although it typically affects menopausal and postmenopausal women, it can cause comorbidities in women of any age. In fact, nearly 20,000 new cases and 14,000 deaths are projected by the American Cancer Society just for the United States of America during 2023 [170]. If identified in the early stages, ovarian cancer presents a very high rate of successful cure, hence, physicians and researchers are constantly searching for newer, more accurate, and more rapid procedures to diagnose the pathology. The identification of specific VOCs in women’s breath as a biomarker for disease diagnosis has started to garner attention [171,172].

Some work has been carried out regarding urinary VOCs. Niemi et al. (2018), for example, analysed the VOCs emitted in urinary samples of ovarian cancer patients aiming to differentiate between this group and healthy individuals. A field asymmetric ion mobility spectrometry (FAIMS) device was used to analyse the headspace formed by the samples of urine previously collected from healthy women (18 individuals) and ovarian cancer patients, including malignant and benign tumours (51 individuals). The specific patterns of VOCs detected by FAIMS enabled the authors to distinguish between healthy individuals from malignant tumour patients with accuracy, sensitivity, and specificity levels of 81.3%, 91.2%, and 63.1%, respectively. Considering the malignant and benign tumours, the patients of both these groups were distinguished with accuracy, sensitivity, and specificity levels of 77.3%, 91.5% and 51.4%, respectively [173].

A few studies have been developed regarding the volatile organic compounds produced by biological processes related to ovarian cancer and emitted in the exhaled breath of patients. Amal et al. (2015), for example, developed a pilot study that gathered a cohort of 182 women (48 ovarian cancer patients, 48 healthy individuals, and 86 benign gynaecological neoplasia), and analysed their exhaled breath on a GC-MS device. By considering the specific breath signatures of each group, the authors were able to discriminate between ovarian cancer patients and healthy individuals with accuracy, sensitivity, and specificity levels of 89%, 79%, and 100%, respectively [174]. Kahn et al. (2015), in their turn, were able to differentiate healthy women from ovarian cancer patients through their exhaled breath patterns with an accuracy level of 82%. To achieve such results, the authors developed flexible sensors based on modified gold nanoparticles and integrated them into a dynamic cross-reactive diagnostic sensing array to detect the volatile organic compounds exhaled in the breath of the volunteers. In addition, the authors identified six VOCs as potential biomarkers for ovarian cancer diagnosis, styrene, nonanal, hexadecane, decanal, 3-heptanone, and 2-ethylhexanol [175]. Finally, Raspagliesi et al. (2020), in a more recent study, used an electronic nose to separate the analytes of breath samples from 251 female volunteers (114 healthy individuals, 51 benign tumour patients, and 86 ovarian cancer patients). It is worth mentioning that the e-nose used by the authors contains 10 types of sensors identified as S1 (aromatic compounds), S2 (broad-range compounds, polar compounds, nitrogen oxides, and ozone), S3 (ammonia, aromatic compounds, aldehydes, and ketones), S4 (hydrogen), S5 (alkanes, aromatic compounds, and less polar compounds), S6 (methane and broad-range compounds), S7 (sulphur compounds, terpenes, and sulphur organic compounds), S8 (alcohols, partially aromatic compounds, and ketones), S9 (aromatic compounds and sulphur organic compounds), and S10 (methane), and responsible for the detection of specific categories of compounds. The differentiation of ovarian cancer patients from healthy women based on their respective VOC patterns was achieved with sensitivity and specificity levels of 98% and 95%, respectively. When considering the differentiation of ovarian cancer patients from the group formed by healthy and benign tumour volunteers, the sensitivity and specificity levels change to 89% and 86% [38]. As already remarked, there is a long but promising path to cover in the field of biomarker VOCs for ovarian cancer diagnosis.

#### 2.7.4. Pancreatic Cancer

Pancreatic cancer is one of the most challenging cancers in terms of diagnosis. Most patients are diagnosed at the late stages of the disease when any chance of cure is already very diminished. Regarding this issue, new technologies that ensure a faster and more accurate identification of the pathology must be explored and studied [176,177].

The volatile organic compounds from urinary samples previously collected from pancreatic cancer patients have been studied as potential biomarkers to diagnose the pathology. However, breath VOCs have not received the same attention [178,179]. The suitability of breath analytes for pancreatic cancer diagnosis was assessed by Markar et al. (2018) [39]. A total of 132 volunteers (57 pancreatic cancer patients and 75 healthy individuals) were used for exhaled breath analysis with a GC-MS device. Among the 66 identified analytes, 12 VOCs (1-butanol, amylene hydrate, tetradecane, undecane, benzaldehyde, acetoin, 1-(methylthio)-propane, n-hexane, isopropyl alcohol, acetone, pentane, and formaldehyde) were remarked as potential biomarkers in the breath [39]. Princivalle et al. (2018), in their turn, were able to use a pattern of 10 VOCs to differentiate between pancreatic patients (65 individuals) and healthy volunteers (102 individuals) with sensitivity and specificity levels of 100% and 84%, respectively. The compounds identified as potential biomarkers for the diagnosis of pancreatic cancer were n-heptane, toluene, benzene, n-propanol, isoprene, acetone, ethanol, acetonitrile, butanone, and methanol [180]. More than two dozen VOCs were proved to be of special interest in the identification of breath biomarkers for pancreatic cancer; therefore, further studies must be developed and deeper knowledge is waiting to be discovered.

#### 2.7.5. Thyroid Cancer

Thyroid cancer is a subtype of Squamous cell carcinoma, commonly known as head and neck cancer (HNC). It is a type of cancer that presents higher gender disparity in terms of incidence and severity, affecting three times more women than men and, due to this fact, is one of the main causes of women’s deaths [181,182]. The necessity of earlier diagnosis has raised the relevance of biomarkers and, specifically, VOC biomarkers, as a tool for rapid, non-invasive, and accurate pathology diagnosis [183]. One of the few studies developed so far on the potentiality of VOCs as biomarkers for thyroid cancer diagnosis was developed by Guo et al. (2015). A cohort of 96 volunteers (32 healthy individuals and 64 thyroid cancer patients) was used for exhaled breath sampling and analysis. The VOCs were detected with a solid-phase microextraction–gas chromatography–mass spectrometry (SPME-GC-MS) device. The authors were able to identify 13 volatile organic compounds whose concentration levels vary with the presence or absence of the pathology; hence, all of them are potential biomarkers of thyroid cancer [40]. As can be seen, the field of VOCs as biomarkers for thyroid cancer is in development and further studies must be developed to explore it accordingly.

### 2.8. Current Limitations in Breath Biomarkers

A major attraction of breath biomarkers and their use for clinical diagnosis is their non-invasiveness and potential for early disease diagnosis. The detection and consequent identification and quantification of the volatile organic compounds from breath has an auspicious and promising future for the diagnostic and treatment optimization through precision medicine. Nonetheless, it is important to summarily address the limitations still existing in this field and the challenges that must be overcome before the use of biomarkers for clinical purposes becomes a reality.

A final aim of the biomarker research reported in the present review is to achieve the certification of the selected volatile organic compounds detected in the breath that can act as biomarkers for the diagnosis of the mentioned oncological pathologies. However, the certification of new procedures and methodologies for medical use is a time-consuming and complex route that implies further studies, multiple experiments, and totally safe and reproducible results. Presently, only a few volatile compounds, such as ethanol, hydrogen, nitric oxide carbon monoxide, and branched hydrocarbons, have been approved by regulatory institutions and successfully recognized and accepted as biomarkers of specific diseases; however, none of them are for carcinogenic pathologies.

In general, the obstacles in the certification of potential VOC biomarkers that limit their passage into clinical practice are linked to the biological nature of the composition of exhaled air and the technical issues related to their detection, analysis, and validation.

Firstly, it is important to draw attention to the fact that all potential VOC biomarkers of cancer are present at a very low concentration range (ppb_v_ to ppt_v_). Therefore, reliable collection procedures of breath samples and accurate detection of VOCs are essential to identify suitable biomarkers. As evident throughout the reviewed documents, there is no certified equipment nor a standardised methodology that enables the collection of exhaled air samples. Most of the sampling procedures used fail to fully isolate the breath samples from exogenous contaminants, as well as to identify the origins of endogenous VOCs. Additionally, the storage conditions of collected breath samples and the influence of external factors (temperature and humidity) may lead to the degradation of the samples and, consequently, to inaccurate results. For this reason, the certification and standardization of sampling methodologies, and the study of the biological origins of target VOCs are required and necessary tasks for the clear association of potential VOC of cancer with relevant metabolic changes.

Current analytical techniques used to analyse and identify volatile organic compounds from breath also need additional validation in order to meet regulatory requirements. Some of the techniques used for reported data fail to achieve detection limits as low as the concentrations of VOCs commonly found in breath. Others do not have the required sensitivity or selectivity to identify all analytes present in complex matrices such as those found in the breath. Therefore, the lack of standardization in analytical procedures and different methodological approaches limit the possibility of comparing and combining the data reported. Also, the lack of portability and complexity of the hardware presently used as analytical equipment are other limitations for on-site clinical analysis and large clinical trials.

Finally, an important challenge in the certification of potential VOC biomarkers for cancer is linked to the lack of specificity of the compounds themselves. As is evident from the tables included throughout this review, there is no single specific volatile compound related to a specific oncological disease, but rather several VOCs are reported for each kind of cancer. On the other hand, a considerably elevated number of analytes have been reported as directly relatable to more than one health condition. One example is acetone, which has been studied regarding its direct relationship with at least three carcinogenic conditions, namely lung cancer, gastric cancer, and colorectal cancer.

In summary, the reported data on potential VOC biomarkers of cancer present promising results and a considerable amount of research that is under development at the moment. However, the field faces major challenges in the standardization of breath sampling and analytical procedures, as well as data interpretation. Rigorous study design and large clinical trials need to be implemented before the breath analysis and VOC biomarkers of cancer can finally be certified for medical applications and clinical purposes.

## 3. Conclusions

The qualification and quantification of endogenous volatile organic compounds through exhaled breath as biomarkers to diagnose several forms of carcinogenic pathologies have been gaining relevance in recent decades. New analytes are often identified and related to the development of cancer, and their assessment can lead to a rapid, accurate, painless, non-invasive, and cheap diagnosis procedure. Therefore, the present paper has addressed the enormous potential of VOCs as breath biomarkers by gathering the main analytes already identified in the scientific literature as carcinogenic biomarkers.

A total of 265 putative biomarkers were addressed throughout the paper as biomarkers for the identification of breast, colorectal, gastric, lung, prostate, and squamous cell cancers. The most relevant bibliographic sources were reviewed and future perspectives regarding new findings in the field of exhaled VOCs for pathological diagnosis were discussed. Considering all the reviewed works, the hundreds of analytes addressed, and the future perspectives, it is safe to state that the field of VOCs as breath biomarkers represents a promising and extremely relevant method of diagnosis of carcinogenic conditions.

On the other hand, it is important to mention that the implementation of breath VOC analysis in medical practice is only possible after the validation of the volatile organic biomarkers by regulatory institutions. Since VOC biomarkers are not very abundant in exhaled air (<1% compared to other compounds) and present trace concentrations (in the order of low ppm_v_ to ppt_v_), it is essential to implement reliable and repeatable methodologies for sample collection, and standardized procedures to accurately identify VOC biomarkers.

## Figures and Tables

**Table 1:** Summary of all the reviewed breath biomarker VOCs for the diagnosis of lung cancer.

Volatile Organic Compounds	CAS Numbers	HMDB Numbers	References
Acetone	67-64-1	HMDB0001659	[50,67,68,70]
Benzene	71-43-2	HMDB0001505	[50,67]
Butanal	123-72-8	HMDB0003543	[52,65,67]
Butane	106-97-8	---	[72]
2-Butanone	78-93-3	HMDB0000474	[50,54,67,68,69]
Carbon disulphide	75-15-0	HMDB0036574	[66,67]
Chloroform	67-66-3	HMDB0029596	[50]
Cyclohexane	110-82-7	HMDB0029597	[50,53]
Decane	124-18-5	HMDB0031450	[50]
2,4-Dimethylheptane	2213-23-2	---	[72]
Dimethyl sulphide	75-18-3	HMDB0002303	[68]
Ethanol	64-17-5	HMDB0000108	[50,68]
Ethylbenzene	100-41-4	HMDB0059905	[54,66,67]
Furan	110-00-9	HMDB0013785	[67]
Heptanal	111-71-7	HMDB0031475	[52,65]
Hexanal	66-25-1	HMDB0005994	[51,52,54,64,65]
Hexene	592-41-6	---	[50]
2-Hydroxy acetaldehyde	141-46-8	HMDB0003344	[69]
3-Hydroxy-2-butanone	513-86-0	HMDB0003243	[69]
4-Hydroxyhexenal	17427-21-3	---	[69]
Isoprene	78-79-5	HMDB0253673	[50,51,54,70]
Isopropanol	67-63-0	HMDB0000863	[50,66,67,68]
Methanol	67-56-1	HMDB0001875	[50,70]
2-Methylbutane	78-78-4	HMDB0253668	[72]
4-Methyloctane	2216-34-4	---	[72]
Nonanal	124-19-6	HMDB0059835	[52,64,65]
Octanal	124-13-0	HMDB0001140	[52,64,65,73]
Pentanal	110-62-3	HMDB0031206	[52,64,65]
Pentane	109-66-0	HMDB0029603	[70]
2-Pentanone	107-87-9	HMDB0034235	[67,68,72]
Propanal	123-38-6	HMDB0003366	[65,67,72]
Propane	74-98-6	HMDB0031630	[50,72]
Propanol	71-23-8	HMDB0000820	[50,53,54,67,68,70]
2-Propenal	107-02-8	HMDB0041822	[66,67]
Propene	115-07-1	HMDB0256839	[66,72]
Styrene	100-42-5	HMDB0034240	[50,51,54]
Toluene	108-88-3	HMDB0034168	[50,53]
1,2,4-Trimethylbenzene	95-63-6	HMDB0013733	[50]
o-Xylene	95-47-6	HMDB0059851	[50,53]
p-Xylene	106-42-3	HMDB0059924	[51]

**Table 2:** Summary of all the reviewed breath biomarker VOCs for the diagnosis of breast cancer.

Volatile Organic Compounds	CAS Numbers	HMDB Numbers	References
Acetic Acid	64-19-7	HMDB0000042	[84]
Acetophenone	98-86-2	HMDB0033910	[97]
(+)-Aromadendrene	489-39-4	---	[95]
Benzaldehyde	100-52-7	HMDB0006115	[85]
2-Butyloctanol	3913-02-8	HMDB0041288	[95]
Caryophyllene	87-44-5	HMDB0036792	[96]
Cyclohexanol	108-93-0	---	[85,98]
Cyclohexanone	108-94-1	HMDB0003315	[99]
Cyclopentane	287-92-3	---	[84]
Cyclopentanone	120-92-3	HMDB0031407	[99]
Decene	872-05-9	---	[96]
2,4-Dimethylbenzaldehyde	15764-16-6	HMDB0032142	[85,98]
1,3-Dimethylbenzene	108-38-3	HMDB0059810	[84]
2,2-Dimethylbutane	75-83-2	HMDB0245332	[85]
1,4-Dimethylcyclohexane	589-90-2	---	[84]
2,3-Dimethylhexane	584-94-1	HMDB0037617	[85]
2,6-Dimethyloctane	2051-30-1	---	[99]
2,4-Dimethylpentane	108-08-7	HMDB0245455	[84]
1,3-Di-ter-butylbenzene	1014-60-4	HMDB0061923	[85]
2,5-Ditert-butylcyclohexa-2,5-diene-1,4-dione	2460-77-7	---	[95]
2,6-Ditert-butylcyclohexa-2,5-diene-1,4-dione	719-22-2	HMDB0013817	[95]
Dodecane	112-40-3	HMDB0031444	[95]
2-Dodecanone	6175-49-1	HMDB0031019	[85]
Ethanol	64-17-5	HMDB0059905	[84]
Ethyl acetate	141-78-6	HMDB0031217	[87]
1-Ethyl-3,5-dimethylbenzene	934-74-7	---	[95]
Ethylene Carbonate	96-49-1	HMDB0252067	[99]
Ethylidenecyclopropane	18631-83-9	---	[95]
2-Ethylhexanol	104-76-7	HMDB0031231	[84,98,99]
Ethyl propanoate	105-37-3	HMDB0030058	[87]
Ethyl-Tris(Trimethylsilyl)-Silicate	18030-67-6	---	[95]
Heptanal	111-71-7	HMDB0031475	[97]
Heptane	142-82-5	HMDB0031447	[84,85]
2-Heptanone	110-43-0	HMDB0003671	[87]
Hexamethyldisilane	1450-14-2	---	[99]
2-Hexyloctanol	19780-79-1	---	[95]
Isobutyric acid	79-31-2	HMDB0001873	[85]
Isoprene	78-79-5	HMDB0253673	[95]
Isopropanol	67-63-0	HMDB0000863	[97]
Isopropylmyristate	110-27-0	HMDB0040392	[97]
Limonene	138-86-3	HMDB0003375	[95,100]
(+)-Longifolene	475-20-7	HMDB0302687	[95]
Menthol	89-78-1	HMDB0003352	[99]
Methanol	67-56-1	HMDB0001875	[86]
3-Methoxy-1,2-propanediol	623-39-2	---	[99]
Methylacrylic acid	79-41-4	HMDB0254514	[99]
2-Methyl-1,2-bis(trimethylsiloxy)-propane	6651-34-9	---	[99]
2-Methylbutanoic acid	116-53-0	HMDB0002176	[87]
3-Methyl-3-butenol	763-32-6	HMDB0030126	[87]
4-Methyl-2-heptanone	6137-06-0	HMDB0013821	[85]
6-Methyl-5-hepten-2-one	110-93-0	HMDB0035915	[84]
3-Methylhexane	589-34-4	HMDB0245932	[96]
(R)-1-Methyl-5-(1-methyl)cyclohexene	1461-27-4	---	[95]
3-Methylpyridine	108-99-6	HMDB0061887	[99]
Naphthalene	91-20-3	HMDB0029751	[96]
2-Nonanone	821-55-6	HMDB0031266	[85]
Octamethylcyclotetrasiloxane	556-67-2	---	[95]
Pentadecane	629-62-9	HMDB0059886	[95]
1,4-Pentadiene	591-93-5	---	[95]
2-Pentanone	107-87-9	HMDB0034235	[87]
2-Phenyl-2-propanol	617-94-7	---	[99]
Phenol	108-95-2	HMDB0000228	[99]
α-Pinene	80-56-8	HMDB0006525	[84]
1,2-Propanediol	57-55-6	HMDB0001881	[99]
2-Propenoic acid	79-10-7	HMDB0031647	[84]
Pyrrolidine	123-75-1	HMDB0031641	[85]
Tetradecane	629-59-4	HMDB0059907	[95]
1,2,3,5-Tetramethylbenzene	527-53-7	HMDB0244133	[95]
1,2,4,5-Tetramethylbenzene	95-93-2	HMDB0244147	[95]
Tetramethylsilicane	75-76-3	---	[99]
1,1,3,3-Tetramethylurea	632-22-4	HMDB0062789	[99]
Toluene	108-88-3	HMDB0034168	[84]
Trichlorethylene	79-01-6	HMDB0029593	[96]
Tridecane	629-50-5	HMDB0034284	[95]
Trifluoroacetic acid	76-05-1	HMDB0034284	[95]
2,6,11-Trimethyldodecane	31295-56-4	HMDB0302691	[95]
2,7,10-Trimethyldodecane	74645-98-0	HMDB0062790	[95]
Undecane	1120-21-4	HMDB0031445	[95]
p-Xylene	106-42-3	HMDB0059924	[85,98]

**Table 3:** Summary of all the reviewed breath biomarker VOCs for the diagnosis of gastric cancer.

Volatile Organic Compounds	CAS Numbers	HMDB Numbers	References
Acetic Acid	64-19-7	HMDB0000042	[110]
Aceticamide	60-35-5	HMDB0031645	[110]
Acetone	67-64-1	HMDB0001659	[111,112]
1,4-Butanediol	110-63-4	HMDB0244201	[107]
2-Butanone	78-93-3	HMDB0000474	[107,109]
4-Butoxybutanol	4161-24-4	---	[107]
2-Butoxyethanol	111-76-2	HMDB0031327	[108,109]
2,3-Dimethylpentane	565-59-3	---	[112]
1,3-Dioxolan-2-one	96-49-1	HMDB0252067	[111]
Dodecane	112-40-3	HMDB0031444	[112]
Ethylene	74-85-1	HMDB0029594	[110]
Formic acid propylester	110-74-7	HMDB0040253	[107]
Furfural	98-01-1	HMDB0032914	[108,109]
Hexane	110-54-3	HMDB0029600	[112]
Hexanol	111-27-3	HMDB0012971	[112]
Isoprene	78-79-5	HMDB0253673	[108,110,111,112]
4-Isopropoxylbutanol	42042-71-7	---	[107]
Menthol	89-78-1	HMDB0003352	[112]
6-Methyl-5-hepten-2-one	110-93-0	HMDB0035915	[108]
2-Methylhexane	591-76-4	HMDB0245230	[112]
3-Methylhexane	589-34-4	HMDB0245932	[112]
Methylisobutylketone	108-10-1	HMDB0002939	[110]
4-Methyloctane	2216-34-4	---	[109]
2-Methylpentane	107-83-5	HMDB0061884	[112]
3-Methylpentane	96-14-0	HMDB0061885	[112]
α-Methylstyrene	98-83-9	HMDB0059899	[109]
Nonanol	28473-21-4	HMDB0031265	[107]
3-Octanone	106-68-3	---	[107]
Phenol	108-95-2	HMDB0000228	[111]
Phenyl acetate	122-79-2	HMDB0040733	[111,112]
Pivalic acid	75-98-9	HMDB0041992	[112]
Propanal	123-38-6	HMDB0003366	[110]
1,3-Propanediol	504-63-2	---	[110]
2-Propenenitrile	107-13-1	HMDB0247972	[108,109]
Tetradecane	629-59-4	HMDB0059907	[112]
Tolualdehyde	1334-78-7	HMDB0006236	[110]
1,2,3-Trimethylbenzene	526-73-8	HMDB0059901	[109,111]
1,3,5-Trimethylbenzene	108-67-8	HMDB0041924	[110]
2,6,11-Trimethyldodecane	31295-56-4	HMDB0302691	[107]
m-Xylene	108-38-3	HMDB0059810	[111]

**Table 4:** Summary of all the reviewed breath biomarker VOCs for the diagnosis of colorectal cancer.

Volatile Organic Compounds	CAS Numbers	HMDB Numbers	References
Acetone	67-64-1	HMDB0001659	[121]
Benzaldehyde	100-52-7	HMDB0006115	[120,123]
Benzoic acid	65-85-0	HMDB0001870	[120]
Butanoic acid	107-92-6	HMDB0000039	[123]
Butylatedhydroxytoluene	128-37-0	HMDB0033826	[120]
6-t-Butyl-2,2,9,9-tetramethyl-3,5-decadien-7-yne	---	---	[31]
Cyclohexane	110-82-7	HMDB0029597	[118,119]
Cyclohexanone	108-94-1	HMDB0003315	[31,119]
Cyclooctylmethanol	3637-63-6	---	[31]
Decanal	112-31-2	HMDB0011623	[118,119]
1,3-Dimethylbenzene	108-38-3	HMDB0059810	[118,119]
1,4-Dimethylbenzene	106-42-3	HMDB0059924	[118,119]
2,2-Dimethyldecane	17302-37-3	HMDB0302690	[31]
6,10-Dimethyl-5,9-undecadien-2-one	689-67-8	HMDB0031846	[120]
Dodecane	112-40-3	HMDB0031444	[31,120]
Dodecanoic acid	143-07-7	HMDB0000638	[123]
Ethanol	64-17-5	HMDB0000108	[121]
Ethyl acetate	141-78-6	HMDB0031217	[121]
Ethylaniline	103-69-5	HMDB0302429	[31]
Ethylbenzene	100-41-4	HMDB0059905	[120,123]
4-Ethyl-1-octyn-3-ol	5877-42-9	---	[31]
3-Hydroxy-2,4,4-trimethylpentyl 2-methylpropanoate	74367-34-3	---	[31]
Indole	120-72-9	HMDB0000738	[123]
Methylbenzene	108-88-3	HMDB0034168	[120,123]
2-Methylbutane	78-78-4	HMDB0253668	[118,119]
Methylcyclohexane	108-87-2	---	[118,119]
Methylcyclopentane	96-37-7	HMDB0031542	[118,119]
4-Methyloctane	2216-34-4	---	[118,121]
2-Methylpentane	107-83-5	HMDB0061884	[118]
3-Methylpentane	96-14-0	HMDB0061885	[118,119]
4-methyl-2-pentanone	108-10-1	HMDB0002939	[118,119]
4-Methylundecane	2980-69-0	---	[118]
Nonanal	124-19-6	HMDB0059835	[118,119,123]
Octanoic acid	124-07-2	HMDB0040195	[123]
1,2-Pentadiene	591-95-7	---	[118,119]
Pentanoic acid	109-52-4	HMDB0000892	[123]
Phenol	108-95-2	HMDB0000228	[123]
Tetradecane	629-59-4	HMDB0059907	[120,123]
Trans-2-dodecenol	69064-37-5	---	[31]
Tridecane	629-50-5	HMDB0034284	[120]
Trimethyldecane	98060-54-9	---	[118]

**Table 5:** Summary of all the reviewed breath biomarker VOCs for the diagnosis of prostate cancer.

Volatile Organic Compounds	CAS Numbers	HMDB Numbers	References
Acetaldehyde	75-07-0	HMDB0000990	[131]
Acetyl acetate	108-24-7	HMDB0031646	[131]
Ethyl butyrate	105-54-4	HMDB0033889	[131]
Ethyl vinyl ketone	1629-58-9	HMDB0031607	[131]
Methyl butyrate	623-42-7	HMDB0033890	[131]
Propyl propionate	106-36-5	HMDB0030059	[131]

**Table 6:** Summary of all the reviewed breath biomarker VOCs for the diagnosis of squamous cell cancer.

Volatile Organic Compounds	CAS Numbers	HMDB Numbers	References
Acetophenone	98-86-2	HMDB0033910	[143]
Ammonium acetate	631-61-8	---	[144]
Benzaldehyde	100-52-7	HMDB0006115	[134]
2,5-Bis-1,1-dimethylethylphenol	5875-45-6	---	[145]
2,3-Butanediol	513-85-9	HMDB0003156	[152]
2,3-Butanedione	431-03-8	HMDB0003407	[143]
2-Butanone	78-93-3	HMDB0000474	[152]
Butyric Acid	107-92-6	HMDB0000039	[153]
Decanal	112-31-2	HMDB0011623	[134]
1,2-Decanediol	1119-86-4	---	[145]
E-3-Decen-2-ol	18402-84-1	HMDB0013810	[145]
1,4-Dichlorobenzene	106-46-7	HMDB0041971	[145]
Dihydro-2(3H)-furanone	96-48-0	HMDB0000549	[143]
2,2-Dimethylbutane	75-83-2	HMDB0245332	[143]
2,3-Dimethylbutane	79-29-8	HMDB0245401	[143]
2,2-Dimethyldecane	17302-37-3	---	[144]
4,6-Dimethyl-dodecane	61141-72-8	---	[144]
2,4-Dimethylheptane	2213-23-2	HMDB0031416	[144]
4,5-Dimethylnonane	17302-23-7	---	[134]
2,2-Dimethylpropanoic acid	75-98-9	HMDB0041992	[144]
3,7-Dimethylundecane	17301-29-0	---	[134]
Dodecane	112-40-3	HMDB0031444	[134]
Ethanol	64-17-5	HMDB0000108	[147,152]
Ethylphenol	90-00-6	HMDB0302562	[158]
2-Ethyltoluene	611-14-3	HMDB0059819	[143]
3-Ethyltoluene	620-14-4	HMDB0059848	[143]
4-Ethyltoluene	622-96-8	HMDB0059832	[143]
Heptanoic Acid	111-14-8	HMDB0000666	[153]
Hexadecane	544-76-3	HMDB0033792	[134]
Hexanoic Acid	142-62-1	HMDB0000535	[153,158]
Hydrogen cyanide	74-90-8	HMDB0060292	[146]
Limonene	138-86-3	HMDB0003375	[144]
2-Methylbutanal	96-17-3	HMDB0031526	[143]
3-Methylbutanal	590-86-3	HMDB0006478	[143]
3-Methyl-2-butanone	563-80-4	---	[143]
3-Methylhexane	589-34-4	HMDB0245932	[144]
5-Methyl-3-hexanone	623-56-3	HMDB0031549	[144]
3-Methylnonane	5911-04-6	---	[144]
4-Methyloctane	2216-34-4	---	[144]
2-Methylpentane	107-83-5	HMDB0061884	[143]
3-Methylpentane	96-14-0	HMDB0061885	[143]
Methylphenol	620-17-7	---	[158]
2-Methyltetrahydrofuran	96-47-9	HMDB0245240	[143]
Octene	111-66-0	HMDB0032449	[134]
Pentanal	110-62-3	HMDB0031206	[143]
Pentanoic acid	109-52-4	HMDB0000892	[153]
2-Pentanone	107-87-9	HMDB0034235	[143]
Phenol	108-95-2	HMDB0000228	[158]
2-Propenenitrile	107-13-1	HMDB0247972	[147]
9-Tetradecenol	52957-16-1	---	[152]
Toluene	108-88-3	HMDB0034168	[143]
Triglyceride	32765-69-8	HMDB0005474	[143]
Trimethylamine	75-50-3	HMDB0000906	[143]
1,2,3-Trimethylbenzene	526-73-8	HMDB0059901	[143]
1,2,4-Trimethylbenzene	95-63-6	HMDB0013733	[143]
1,3,5-Trimethylbenzene	108-67-8	HMDB0041924	[143]
2,6,6-Trimethyloctane	54166-32-4	---	[144]
Undecane	1120-21-4	HMDB0031445	[134,147]
p-Xylene	106-42-3	HMDB0059924	[144]

## Data Availability

No data were created during this study.
